# The Effect of Labor and Relationship Exclusions on Older Korean Men with Depression

**DOI:** 10.3390/ijerph18115876

**Published:** 2021-05-30

**Authors:** Eun-Jung Kwon, Hye-Sun Jung

**Affiliations:** Department of Preventive Medicine, College of Medicine, The Catholic University of Korea, 222 Banpo-daero, Seocho-gu, Seoul 06591, Korea; kwonruby@naver.com

**Keywords:** labor exclusion, relationship exclusion, depression, the Korean Longitudinal Study of Aging

## Abstract

To manage depression of older men, it is necessary to identify factors that influence depression and improve them. This study used the Korean Aging Longitude Research to understand the effects of labor exclusion and relationship exclusion on depression in Korean male seniors aged 65 and older. According to research on the effect of labor exclusion and relationship exclusion on depression, depression in the case where labor was excluded was 1.69 times higher in 2014, 1.65 times higher in 2016, and 1.93 times higher in 2018 compared to the case where labor was not excluded. Depression in the case where relationship was excluded was 2.94 times higher in 2014, 3.15 times higher in 2016, and 2.57 times higher in 2018 compared to the case where relationship was not excluded. Depression in the case where labor and relationship were both excluded was 3.00 times higher in 2014, 3.23 times higher in 2016, and 2.81 times higher in 2018 compared to the case where neither labor nor relationship was excluded. Since labor exclusion and relationship exclusion have a big influence on depression in older men, it is necessary to establish plans for job creation and for the formation of social relationships for the elderly.

## 1. Introduction

As the Korean population is aging, depression in the elderly is emerging as an important health issue. According to the standards set by the United Nations (UN), if over 7% of an entire population is 65 years or older, it is categorized as an aging society; if this is more than 14%, it is an aged society, while a proportion more than 20% constitutes a super-aged society [1]. In 2018, Korea became an aged society, as the aging population accounted for 14.29% [2]. The proportion of the aging population in Korea in 2020 was 15.7% [3]. Aging occurs at a high rate in Korea, as it took 18 years [2] for Korea to become an aged society from previously being an aging society. Japan, one of the countries with a rapid aging rate, took 24 years to enter an aged society from a hitherto aging society.

The elderly are subject to a variety of negative incidents and experiences, such as deterioration of physical functioning, retirement, and loss of a spouse, which makes them more vulnerable to depression than other age groups; it is difficult for them to overcome these factors [4]. Not only does depression in the elderly cause great emotional pain, but it also degrades their quality of life. Depression is one of the most important causes of suicide in the elderly [5]. Since the suicide rate of the elderly in Korea is 53.3 per every 100,000 people (ranking first in the Organization for Economic Cooperation and Development (OECD) member nations and 2.9 times higher than the OECD national average), it is an important issue that should be solved on a national scale [6].

Various factors influence depression in the elderly. According to previous research, the incidence of depression increased with bereavement [7], a low level of education [8], residing in a small city [9], religion [10], a low household income [8], smoking [11], a lack of physical activity [12,13,14], and having a chronic illness [11,15]. On the other hand, research suggests that a greater engagement in social relationships and social activities among the elderly leads to lower depression levels [16,17]. Therefore, it is necessary to manage the deteriorating factors and social exclusion to lower the incidence of depression in the elderly.

Social exclusion is a concept that is opposite to social integration. It refers to the process of being entirely or partly excluded from the society to which one belongs [18]. Types of social exclusion include isolation concepts in various aspects, such as housing, education, employment, health services, law and politics, and social network exclusions [18]. Social exclusion of the elderly is likely to produce more complex and cumulative problems than other age groups because of life events, such as retirement, economic plights, and loss of spouse, among other factors [19]. If the older adults are socially excluded, social difficulties increase as their inequality and disadvantages within society are aggravated, and they are likely to lose their support network and be isolated due to a reduced social network [19].

Elderly people who are excluded from the labor market are not only subject to financial pain caused by a decline or loss of income, but also psychosocial problems from loss of their previous roles, a drop in social position, and reduced social relationships [20]. Since the social network of most men is formed around their economic activities, a decline in social networks is greater in retired elderly men than in elderly women [21]. As a result, the labor exclusion of elderly men is closely related to relationship exclusion. If one does not work and cannot maintain social relationships, it may cause physical and mental health issues. According to Ah-Young Lee [22], if older men do not work after retirement, their chances of becoming depressed are two or more times higher than that of elderly women. However, very few studies have examined the association between labor exclusion and depression in elderly men.

Therefore, this study aimed to examine the level of labor and relationship exclusions among elderly men and to identify the influence that labor and relationship exclusions exert on depression.

## 2. Materials and Methods

### 2.1. Study Population

**Figure 1:**
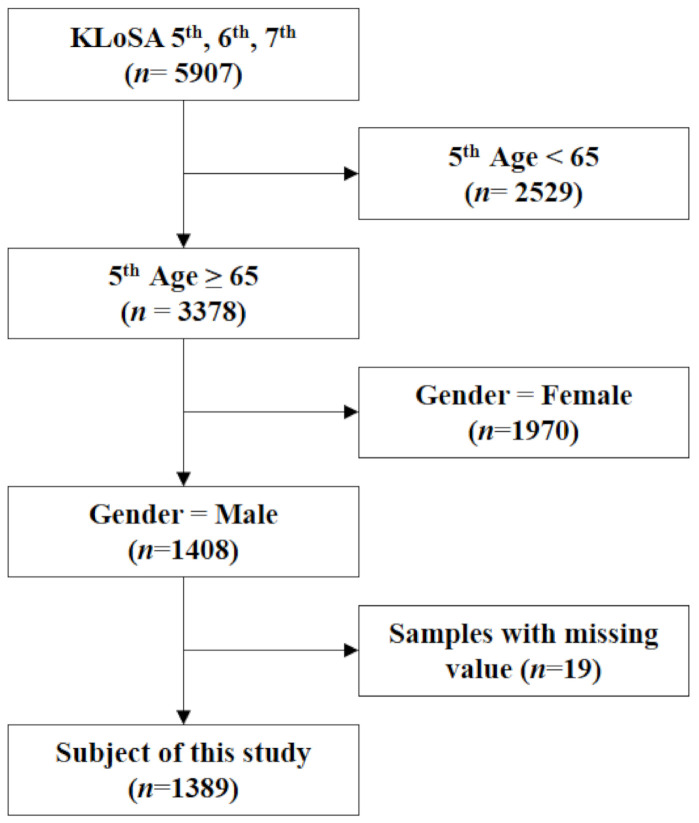
Flow chart of patient selection in this study.

Korean Longitudinal Study of Aging (KLoSA) is a panel survey conducted by the Korean Labor Institute and the Korea Employment Institute Information Service [23]. The survey began in 2006 to produce basic data that establish and enforce effective socioeconomic policies. The first survey was conducted with 10,254 people from 6171 households. Since then, a survey has been conducted every 2 years. The fifth survey was conducted in 2014, the sixth in 2016, and the seventh in 2018. For this study, we selected 5907 people who participated in the fifth, sixth, and seventh KLoSA. Among them, 1389 men were included, excluding people with the following conditions: under the age of 65 years (*n* = 2529), women (*n* = 1970), or lacking data (*n* = 19). As of 2014, the average age of the survey group was 73.26 ± 5.95 (Figure 1).

### 2.2. Measurements and Variable Definitions

#### 2.2.1. General Characteristics

Data on general characteristics, such as the presence or absence of a spouse, educational level, area of residence, religion, gross household income, smoking, drinking, physical activity, and chronic illness were obtained.

As for the presence or absence of a spouse, the subjects were divided into those who had a spouse at the time and those who did not. Those who did not have a spouse were single, bereaved, or divorced. The level of education was classified as elementary-school graduation or lower, middle-school graduation, and high-school graduation or higher. The area of residence was classified into towns, small and medium-sized cities, and large cities. As for religion, the subjects were classified into those who had a religion and those who did not. Gross household income was classified into four groups (<10,000, 10,000–19,999, 20,000–29,999, and ≥30,000 USD). As for smoking, the subjects were classified into those who smoked at the time and those who did not. As for drinking, the subjects were classified into those who drank at the time and those who did not. As for physical activity, those who exercised once or more per week were categorized as “yes”, and those who did not were categorized as “no.” As for chronic illness, those who had at least one of the following illnesses were categorized as “yes”, and those who did not were categorized as “no”: high blood pressure, cerebrovascular disease, or arthritis.

#### 2.2.2. Labor Exclusion and Relationship Exclusion

As for labor exclusion, those who answered “no” to the KLoSA question, “Are you currently working for the purpose of earning an income?”, were defined as those enduring labor exclusions. 

As for relationship exclusion, the KLoSA question, “Do you have any close friends, relatives, or neighbors? If you do, how often do you meet them?”, was asked. Those who answered “not at all” or “less than once every 3–4 months” were defined as those enduring relationship exclusions.

#### 2.2.3. Depression

The degree of depression was assessed using the Korean version of the Center for Epidemiologic Studies Depression Scale (CES-D 10), a test scale that screens for depressive symptoms. This is a simplified version of the CES-D, consisting of 10 questions. According to the standard of Korea’s National Center for Mental Health, the standard for the assessment of depression categorizes 0–2 points as the normal range and 3–10 points as depression [24]. Cronbach’s α from this study was 0.681, 0.639, and 0.719 for the fifth, sixth, and seventh surveys, respectively.

### 2.3. Statistical Analysis

Frequency analysis was conducted to identify the general characteristics of the study participants. The χ^2^ test was performed to verify differences in depression on the basis of the characteristics of the study subjects. Repeated-measures ANOVA was performed to identify the effect of labor and relationship exclusions on changes in depression. Confounding variables were adjusted using multiple logistic regression. For labor exclusion, the subjects not exposed to labor exclusion were the reference group; for the relationship exclusion, the subjects not exposed to relationship exclusion were the reference group; lastly, for both labor and relationship exclusion, the subjects not exposed to both conditions were the reference group. All statistical analyses were performed using IBM SPSS Statistics version 25. A significance level of 5% was verified.

### 2.4. Ethical Considerations

This study was conducted after approval by the Institutional Review Board of the Catholic University of Korea (IRB; MC21EASI0002, approval date: 22 January 2021) and performed in accordance with the Declaration of Helsinki. 

## 3. Results

### 3.1. Subject’s Characteristics

Table 1 lists the characteristics of the subjects, as of 2014. Of the subjects, 91.2% had a spouse, and 42.1% had a high-school education or higher. A total of 39.3% resided in big cities, and 65.0% did not have a religion. Regarding gross household income, 36.1% had an income of 10,000 USD or more. At the time, 78.8% did not smoke, 51.2% did not drink, and 59.4% did not engage in physical activity. A total of 61.1% of the subjects had at least one chronic illness. Those excluded from labor were 62.0% in 2014, 67.6% in 2016, and 72.1% in 2018. Those excluded from relationships were 14.3% in 2014, 15.9% in 2016, and 21.1% in 2018. Those excluded from both labor and relationships were 10.7% in 2014, 14.0% in 2016, and 17.3% in 2018. The prevalence of depression was 46.9% in 2014, 44.5% in 2016, and 53.6% in 2018.

### 3.2. Subject’s Characteristics and Depression

Those with an elementary-school graduation level or lower were more depressed than those with middle-school graduation and high-school graduation levels or higher. Those whose gross household income was below 10,000 USD were more depressed. Those who drank were more depressed than those who did not. Those who were not engaged in physical activity were more depressed than those who were.

In 2014 and 2018, those who did not have a religion were more depressed than those who did. In 2018, those who had a chronic illness were more depressed than those who did not. There was no significant difference in the area of residence and smoking status by year. As for labor and relationship exclusions, in 2014, 2016, and 2018, those excluded from labor were more depressed than those who were not. In 2014, 2016, and 2018, those excluded from relationships were more depressed than those who were not. In 2014, 2016, and 2018, those excluded from both labor and relationships were also more depressed than those who were not (Table 2).

### 3.3. The Level of Depression and Exclusions by Year

Table 3 shows the differences in the level of depression caused by labor and relationship exclusions by year. For depression caused by labor exclusion, the depression scores (out of 10) without labor exclusion were 2.36 ± 0.14 in 2014, 2.37 ± 0.15 in 2016, and 2.68 ± 0.16 in 2018. The depression scores with labor exclusion were 3.32 ± 0.14 0.09 in 2014, 3.18 ± 0.09 in 2016, and 3.74 ± 0.09 in 2018, which were higher than for those without labor exclusion. The depression level by year, with or without labor exclusion, was not statistically significant (*p* = 0.364).

For the depression caused by relationship exclusion, mean depression scores (out of 10) for those without relationship exclusion were 2.81 ± 0.08 in 2014, 2.67 ± 0.08 in 2016, and 3.02 ± 0.09 in 2018. The mean depression scores for those with relationship exclusion were 3.96 ± 0.16 in 2014, 4.03 ± 0.16 and 2016, and 5.03 ± 0.17 in 2018, showing a tendency to increase by year. These were higher than for those without relationship exclusion. The depression level by year, with or without relationship exclusion, was statistically significant (*p* < 0.001).

For depression caused by labor and relationship exclusions, the mean depression scores (out of 10) of non-exclusion cases were 2.80 ± 0.08 in 2014, 2.64 ± 0.08 in 2016, and 3.02 ± 0.09 in 2018. The mean depression scores of cases where labor and relationships were both excluded were 4.29 ± 0.18 in 2014, 4.48 ± 0.18 in 2016, and 5.47 ± 0.19 in 2018, showing a tendency to increase by year. These were higher than non-exclusion cases. The depression level by year, with or without labor and relationship exclusions, was statistically significant (*p* = 0.001).

### 3.4. Exclusions and Depression

For the effect of labor exclusion on depression, in cases with labor exclusion, depression was 1.69 times higher in 2014 (95% CI 1.51–1.89), 1.65 times higher in 2016 (95% CI 1.47–1.86), and 1.93 times higher in 2018 (95% CI 1.71–2.19) compared to cases without labor exclusion.

For the effect of relationship exclusion on depression, in cases with relationships exclusion, depression was 2.94 times higher in 2014 (95% CI 2.50–3.45), 3.15 times higher in 2016 (95% CI 2.68–3.71), and 2.57 times higher in 2018 (95% CI 2.20–2.99) compared to cases without relationships exclusion.

For the effect that the exclusion of both labor and relationships has on depression, in cases where both were excluded, depression was 3.00 times higher in 2014 (95% CI 2.49–3.62), 3.23 times higher in 2016 (95% CI 2.69–3.88), and 2.81 times higher in 2018 (95% CI 2.36–3.36) compared to cases where neither labor nor relationships were excluded (Table 4).

## 4. Discussion

This study analyzed the effect of labor and relationship exclusions on depression among elder Korean men, aged 65 years or older. According to an analysis of the effect of labor exclusion on depression, this was 1.69 times higher in 2014, 1.65 times higher in 2016, and 1.93 times higher in 2018 than in cases without labor exclusion. The results of this study are consistent with those of other previous studies. In a study by Eun-Hye Kim et al. [25] on elderly people in Korea, depression was lower in elderly people who worked. This result was observed again in a study by Ji-Na Han et al. [26]. Additionally, in a study by Christ et al. [27], which used NHIS data of the United States, older people who were excluded from labor were found to have a higher level of depression than those who were not excluded from work. Therefore, it can be verified that exclusion from labor influences depression. In addition, labor exclusion was found to increase by year, reaching 62.0% in 2014, 67.6% in 2016, and 72.1% in 2018. The labor exclusion of older men in Japan was 67.5% as of 2017 [28], which is similar to the results of the present study. It is believed that the labor exclusion levels of Korea and Japan are similar because Korea and Japan have many socioeconomic similarities, and population aging is occurring at a rapid rate in Korea, as with Japan.

According to OECD statistics, in 2017, the proportion of labor force participation in elderly men in Sweden was 21.5%, which was lower than that of Korea for the same period (41.5%) [28]. This is because Sweden has a well-established social welfare system, and the nation provides economic support even if elder men do not participate in labor. However, in Korea, since exclusion from labor is related to elderly poverty, labor exclusion has a significant influence on the elderly, unlike in Sweden. The poverty rate of the elderly in Sweden is 11.3%, which is lower than the OECD national average of 14.8% [29]. Since the elderly poverty rate in Korea is 43.8%, which is notably higher than the OECD national average [29], we can predict that the labor exclusion of elderly people and elderly poverty are closely related. As the elderly in Korea may face poverty after retirement due to decreased income caused by labor exclusion, it is understandable that they wish to remain in the labor market to maintain their earned income.

According to an analysis of the effect of relationship exclusion on depression, depression was 2.94 times higher in 2014, 3.15 times higher in 2016, and 2.57 times higher in 2018 than in cases without relationships exclusion. This is similar to the result of a study on the elderly living alone in Korea, where a higher frequency of contact with friends and neighbors led to lower depression [30]. The results are also similar to a study where, within a Spanish population aged 50 or older, those who were excluded from relationships had a higher depression level than those who were not [31]. The relationship exclusion level of the subjects in this study was found to increase by year, reaching 14.3% in 2014, 15.9% in 2016, and 21.1% in 2018. In a study on elder men, aged 65 to 85, who resided in Tokyo, Japan in 2017, their relationship exclusion was found to be 18.8%. The Japanese study had a stricter standard of relationship exclusion [32] than this study, as it was defined as cases where offline and online contact with family and friends occurred less than once a week, and the relationship exclusion was higher than for the subjects of this study. It is believed that relationship exclusion is more serious in Japan because they became an aging society 30 years prior to Korea and have faced the problems of solitude and isolation earlier than Korea. 

In a study using data from the English Longitudinal Study of Aging (ELSA), a survey of the UK population, aged 50 years or older, measured the relationship exclusion of men as 13.7% in 2014, which is similar to the result of this study [33]. The measurement for the relationship exclusion was based on UCLA loneliness scale which comprised three questions: “How often do you feel you lack companionship?”, “How often do you feel isolated from others?”, and “How often do you feel left out?”. Answers to the questions were hardly ever or never (1 point), some of the time (2 points), or often (3 points), and when the sum of the points was higher than 6, the subject was considered lonely. This measurement is different from our definition of the relationship exclusion and, thus, cannot be compared directly.

According to an analysis of the effect of labor and relationship exclusions on depression, this was 3.00 times higher in 2014, 3.23 times higher in 2016, and 2.81 times higher in 2018 compared to the case where neither labor nor relationships were excluded. The results show that the depression level is higher than when labor and relationships are excluded. A study by Sa-Rang Um et al. [34] reported that older men who were excluded from labor were found to have higher depression and a lower level of social contact than those who were not, and that higher social contact was associated with lower levels of depression. In Korea, older men tend to form social relationships as they work; therefore, if labor is excluded, they tend to be excluded from these relationships as well. If labor and relationship exclusions occur simultaneously, their depression amplifies. Therefore, it is necessary to provide support to prevent labor and relationship exclusions from occurring together. The subjects who were excluded from both labor and relationships were 10.7% in 2014, 14.0% in 2016, and 17.3% in 2018, showing the trend of these exclusions increasing annually. According to a 2004–2005 study on the elderly in Europe, by Vozikaki et al., 9.2% of the elderly felt lonely after retirement [35]. Since the study was conducted on both older men and women, it is difficult to compare it directly to this study. In addition, the survey was conducted in the early 2000s, which was approximately 10 years before this study. Nevertheless, the ratio of those who felt lonely was similar to those of the subjects in this 2014 study. This is believed to be because population aging in Europe progressed faster than in Korea, and it can be argued that labor exclusion due to retirement and other factors affects mental health. 

According to an analysis of the interactive effect of annual changes caused by labor and relationship exclusions, labor exclusion did not show a significant difference in annual changes, but relationship exclusion showed a significant difference. There was also a significant difference when both labor and relationship exclusions occurred. The reason that people work is primarily to accomplish their economic goals. Work also allows an individual to accomplish psychosocial goals. Work enables one to belong to an organization, known as the workplace, to be involved in society, form relationships with people in the organization, and be granted a social role as per their job [36]. Depression is amplified if labor and relationship exclusions occur simultaneously, owing to the formation of social relationships through work. Thus, for elder males, maintaining work and social relationships is an important factor in improving mental health. According to a study by Li et al. [37], a significant correlation between labor and health was found in men, but there was no significant correlation in elder women. This is believed to be because one’s job has a greater meaning to elder men. Since work becomes the center of life for most elder men, significant life changes, such as roles after retirement and reduced social networks, appear significant. As a result, emotional difficulties such as solitude and depression are experienced more seriously. Severe depression may lead to suicide. Since the suicide rate of elderly Koreans is significantly high, preventing labor and relationship exclusions may contribute to the prevention of suicide.

The depression levels of the subjects in this study were 46.9% in 2014, 44.5% in 2016, and 53.6% in 2018. Depression scores were higher in cases with labor and relationships exclusion than in cases without it. In particular, the level of depression for male elders tends to increase annually, indicating that depression issues become more serious in older men. In a study by Soon-Dool Chung and Mi-Jung Koo, the depression level of the Korean baby boomer generation, aged 45–53, was 33.5%. The depression level of the pre-elderly, aged 54–64, was 40.3%. The depression level of those aged 65 or older was 59.6%. This shows that the level of depression elevates with age [38]. According to a study by Richard et al., in elder males who were Medicare beneficiaries and resided in New York City, USA, the depression level was 36.2%, which is lower than that of the subjects in this study [39]. A study conducted in Korea found that depression was associated with the population attributable fraction (PAF) of 45.7% for suicidal ideation in the elderly. This percentage was significantly higher than those of other known risk factors: chronic illness, 19.4%; functional impairment, 4.9%; poor social support, 4.2% [40].

Considering the results of this study, to prevent depression in older men, it is necessary to provide plans to prevent social isolation and solve emotional issues through opportunities for participation in continuous productive activities and maintaining social contact after retirement. At the national level, it is also necessary to establish cooperative systems to manage labor and relationships, by arranging a variety of programs to promote job creation and social relationships. If we try to solve social problems faced by the current generation of the elderly, who are aging at a rapid rate, this can contribute significantly to improving their health.

Despite the discoveries and implications mentioned above, this study had several limitations. Firstly, this study was a cross-sectional investigation based on an auto-administered survey and, thus, cannot reveal causal relations. Secondly, this study did not include various social exclusion factors. These include diverse aspects of exclusion, such as education, law, and politics exclusions; further research that includes such factors should be conducted. Lastly, this study only analyzed labor and relationship exclusion in men. Therefore, caution is required when applying the results of this study to the general population.

## 5. Conclusions

This study analyzed the effect of labor and relationship exclusions on depression among elder Korean men who are 65 years or older. The results of this study show that all these exclusions have a significant effect on depression. In particular, the level of depression was high when labor and relationships were excluded. It is necessary to provide elder men with opportunities to participate in productive and social activities so that they are not excluded from labor and relationships. Additionally, cooperative systems should be established and managed so that elder men can participate in the labor market and strengthen a social network and, hence, are not excluded from labor and relationships.

Based on the above research results, the current study makes the following suggestions:

Firstly, since labor and relationship exclusions have a significant influence on depression in older men, it is necessary to establish plans for job creation and the formation of social relationships for the elderly;

Second, it is necessary to promote programs to alleviate depression in older men, in response to the results showing that the prevention and the management of depression in elder males are becoming more prominent as the population ages.

## Figures and Tables

**Table 1:** Sociodemographic characteristics of subjects.

Variables	Categories	2014	2016	2018
Spouse	Have	1267 (91.2)	1251 (90.1)	1228 (88.4)
	Have not	122 (8.8)	138 (9.9)	161 (11.6)
Level of education	Elementary or lower	535 (38.5)	534 (38.4)	534 (38.4)
	Middle-school graduation	269 (19.4)	269 (19.4)	269 (19.4)
	High school or higher	585 (42.1)	586 (42.2)	586 (42.2)
Area of residence	Town	420 (30.2)	418 (30.1)	427 (30.7)
	Small and middle-sized	423 (30.5)	421 (30.3)	426 (30.7)
	Big city	546 (39.3)	550 (39.6)	536 (38.6)
Religion	Have	486 (35.0)	493 (35.5)	441 (31.7)
	Have not	903 (65.0)	896 (64.5)	948 (68.3)
Gross household income (USD)	≥30,000	257 (18.5)	260 (18.7)	252 (18.1)
	20,000–29,999	229 (16.5)	225 (16.2)	236 (17.0)
	10,000–19,999	402 (28.9)	403 (29.0)	399 (28.7)
	<10,000	501 (36.1)	501 (36.1)	502 (36.1)
Smoking	Yes	295 (21.2)	220 (15.8)	166 (12.0)
	No	1094 (78.8)	1169 (84.2)	1223 (88.0)
Drinking	Yes	711 (51.2)	661 (47.6)	569 (41.0)
	No	678 (48.8)	728 (52.4)	820 (59.0)
Physical activity	Yes	564 (40.6)	589 (42.4)	528 (38.0)
	No	825 (59.4)	800 (57.6)	861 (62.0)
Chronic illness	Yes	849 (61.1)	906 (65.2)	956 (68.8)
	No	540 (38.9)	483 (34.8)	433 (31.2)
Labor exclusion	Not excluded	528 (38.0)	450 (32.4)	388 (27.9)
	Excluded	861 (62.0)	939 (67.6)	1001 (72.1)
Relationship exclusion	Not excluded	1191 (85.7)	1168 (84.1)	1096 (78.9)
	Excluded	198 (14.3)	221 (15.9)	293 (21.1)
Labor and relationship exclusion	Excluded from 1 or none	1240 (89.3)	1195 (86.0)	1149 (82.7)
	Excluded from 2	149 (10.7)	194 (14.0)	240 (17.3)
Depression	Normal	738 (53.1)	771 (55.5)	645 (46.4)
	Depression	651 (46.9)	618 (44.5)	744 (53.6)

Note: Numbers in parentheses are percentages.

**Table 2:** Subject characteristics and depression by year.

		2014				2016				2018			
Variables	Categories	Normal	Depression	χ^2^	p *	Normal	Depression	χ^2^	p *	Normal	Depression	χ^2^	p *
Spouse	Have	698 (55.1)	569 (44.9)	22.231	<0.001	721 (57.6)	530 (42.4)	23.052	<0.001	581 (47.3)	647 (52.7)	3.272	0.042
	Have not	40 (32.8)	82 (67.2)			50 (36.2)	88 (63.8)			64 (39.8)	97 (60.2)		
Level of education	Elementary or lower	255 (47.7)	280 (52.3)	11.892	0.003	270 (50.6)	264 (49.4)	8.601	0.014	224 (41.9)	310 (58.1)	7.936	0.019
	Middle-school graduation	144 (53.5)	125 (46.5)			157 (58.4)	112 (41.6)			126 (46.8)	143 (53.2)		
	High school or higher	339 (57.9)	246 (42.1)			344 (58.7)	242 (41.3)			295 (50.3)	291 (49.7)		
Area of residence	Town	208 (49.5)	212 (50.5)	4.325	0.115	248 (59.3)	170 (40.7)	5.503	0.064	191 (44.7)	236 (55.3)	3.603	0.165
	Small and middle-sized	223 (52.7)	200 (47.3)			216 (51.3)	205 (48.7)			188 (44.1)	238 (55.9)		
	Big city	307 (56.2)	239 (43.8)			307 (55.8)	243 (44.2)			266 (49.6)	270 (50.4)		
Religion	Have	279 (57.4)	207 (42.6)	5.488	0.011	283 (57.4)	210 (42.6)	1.113	0.159	235 (53.3)	206 (46.7)	12.196	<0.001
	Have not	459 (50.8)	444 (49.2)			488 (54.5)	408 (45.5)			410 (43.2)	538 (56.8)		
Gross household income (USD)	≥30,000	149 (58.0)	108 (42.0)	14.995	0.002	154 (59.2)	106 (40.8)	23.339	<0.001	136 (54.0)	116 (46.0)	13.819	0.003
	20,000–29,999	139 (60.7)	90 (39.3)			146 (64.9)	79 (35.1)			122 (51.7)	114 (48.3)		
	10,000–19,999	214 (53.2)	188 (46.8)			233 (57.8)	170 (42.2)			179 (44.9)	220 (55.1)		
	<10,000	236 (47.1)	265 (52.9)			238 (47.5)	263 (52.5)			208 (41.4)	294 (58.6)		
Smoking	Yes	153 (51.9)	142 (48.1)	0.242	0.335	134 (60.9)	86 (39.1)	3.088	0.046	73 (44.0)	93 (56.0)	0.459	0.276
	No	585 (53.5)	509 (46.5)			637 (54.5)	532 (45.5)			572 (46.8)	651 (53.2)		
Drinking	Yes	419 (58.9)	292 (41.1)	19.673	<0.001	407 (61.6)	254 (38.4)	18.789	<0.001	292 (51.3)	277 (48.7)	9.235	0.001
	No	319 (47.1)	359 (52.9)			364 (50.0)	364 (50.0)			353 (43.0)	467 (57.0)		
Physical activity	Yes	317 (56.2)	247 (43.8)	3.603	0.033	341 (57.9)	248 (42.1)	2.360	0.069	288 (54.5)	240 (45.5)	22.519	<0.001
	No	421 (51.0)	404 (49.0)			430 (53.8)	370 (46.3)			357 (41.5)	504 (58.5)		
Chronic illness	Yes	443 (52.2)	406 (47.8)	0.796	0.201	491 (54.2)	415 (45.8)	1.820	0.098	421 (44.0)	535 (56.0)	7.094	0.005
	No	295 (54.6)	245 (45.4)			280 (58.0)	203 (42.0)			224 (51.7)	209 (48.3)		
Labor exclusion	Not excluded	322 (61.0)	206 (39.0)	21.095	<0.001	305 (67.8)	145 (32.2)	40.580	<0.001	227 (58.5)	161 (41.5)	31.529	<0.001
	Excluded	416 (48.3)	445 (51.7)			466 (49.6)	473 (50.4)			418 (41.8)	583 (58.2)		
Relationship exclusion	Not excluded	680 (57.1)	511 (42.9)	52.698	<0.001	710 (60.8)	458 (39.2)	82.871	<0.001	573 (52.3)	523 (47.7)	71.359	<0.001
	Excluded	58 (29.3)	140 (70.7)			61 (27.6)	160 (72.4)			72 (24.6)	221 (75.4)		
Labor and relationship exclusion	Excluded from 1 or none	698 (56.3)	542 (43.7)	46.311	<0.001	720 (60.3)	475 (39.7)	77.952	<0.001	591 (51.4)	558 (48.6)	66.831	<0.001
	Excluded from 2	40 (26.8)	109 (73.2)			51 (26.3)	143 (73.7)			54 (22.5)	186 (77.5)		

* Calculated by chi-square test.

**Table 3:** The level of depression caused by labor and relationship exclusions by year.

IV	Source	SS	df	MS	F	p *
Labor exclusion	Labor exclusion	621.259	1	621.259	42.529	<0.001
	Time	21.901	1.954	11.211	2.832	0.060
	Labor exclusion × time	7.796	1.954	3.99	1.008	0.364
Relationship exclusion	Relationship exclusion	1432.462	1	1432.462	102.201	<0.001
	Time	9.287	1.958	4.744	1.21	0.298
	Relationship exclusion × time	84.517	1.958	43.175	11.01	<0.001
Labor and relationship exclusion	Labor and relationship exclusion	1374.75	1	1374.75	97.79	<0.001
	Time	9.597	1.956	4.906	1.247	0.287
	Labor and relationship exclusion × time	55.846	1.956	28.548	7.255	0.001

* Calculated by repeated-measures ANOVA.

**Table 4:** Odds Ratios of Depression by Exclusion.

		2014			2016			2018		
Variables	Categories	OR	95% CI	*p*	OR	95% CI	*p*	OR	95% CI	*p*
Labor exclusion	Crude *	1.67	1.34–2.08	<0.001	2.14	1.69–2.70	<0.001	1.97	1.55–2.45	<0.001
	Adjusted †‡	1.69	1.51–1.89	<0.001	1.65	1.47–1.86	<0.001	1.93	1.71–2.19	<0.001
Relationship exclusion	Crude *	3.21	2.32–4.45	<0.001	4.07	2.96–5.59	<0.001	3.36	2.51–4.50	<0.001
	Adjusted † ‡	2.94	2.50–3.45	<0.001	3.15	2.68–3.71	<0.001	2.57	2.20–2.99	<0.001
Labor and relationship exclusion	Crude *	3.51	2.40–5.13	<0.001	4.25	3.02–5.97	<0.001	3.65	2.64–5.05	<0.001
	Adjusted †‡	3.00	2.49–3.62	<0.001	3.23	2.69–3.88	<0.001	2.81	2.36–3.36	<0.001

* Calculated by simple logistic regression. † Calculated by multiple logistic regression. ‡ Adjusted for the presence or absence of a spouse, level of education, area of residence, gross household income, smoking, drinking, physical activity, and chronic illness.

## Data Availability

Not applicable.
